# Does social distinction contribute to socioeconomic inequalities in diet: the case of ‘superfoods’ consumption

**DOI:** 10.1186/s12966-017-0495-x

**Published:** 2017-03-27

**Authors:** Joost Oude Groeniger, Frank J. van Lenthe, Mariëlle A. Beenackers, Carlijn B. M. Kamphuis

**Affiliations:** 1 Department of Public Health, Erasmus University Medical Centre, PO Box 2040, 3000 CA Rotterdam, The Netherlands; 2 Department of Human Geography and Spatial Planning, Utrecht University, 3508 TC Utrecht, The Netherlands

**Keywords:** Socioeconomic inequalities, Dietary patterns, Superfoods, Distinction, Cultural participation

## Abstract

**Background:**

The key mechanisms underlying socioeconomic inequalities in dietary intake are still poorly understood, hampering the development of interventions. An important, but sparsely mentioned mechanism is that of ‘social distinction’, whereby those in a higher socioeconomic position adopt dietary patterns by which they can distinguish themselves from lower socioeconomic groups. We investigated the importance of distinction as a mechanism, by testing the socioeconomic gradient in the consumption of so-called ‘superfoods’ and the contribution of a well-established indicator of distinction, cultural participation.

**Methods:**

Data from participants (25–75 years) of the 2014 survey of the Dutch population-based GLOBE study were used (*N* = 2812). Multivariable regression models were used to analyse the association between education, income and cultural participation (e.g. visits to museums, opera, theatre, concerts) and the consumption of superfoods (spelt, quinoa and goji berries, chia seeds or wheatgrass).

**Results:**

The consumption of superfoods is far more prevalent among higher socioeconomic groups. Adjusting for cultural participation strongly attenuated the educational and income gradient in superfoods consumption, whereas cultural participation remained strongly associated with superfoods consumption. Those in the highest quintile of cultural participation reported the highest consumption of spelt products (OR = 2.97, 95% CI = 2.10;4.18), quinoa (OR = 3.50, 95% CI = 2.12;5.79) and goji berries, chia seeds or wheatgrass (OR = 2.69, 95% CI = 1.73;4.17).

**Conclusions:**

The associations between socioeconomic position and the consumption of ‘superfoods’ seem to be partially driven by a process of social distinction. These findings suggest that distinction may be an important, but currently neglected mechanism in generating socioeconomic inequalities in dietary intake. It deserves a more prominent role in interventions to reduce these inequalities.

## Background

Dietary intake is greatly socioeconomically patterned, with those in higher socioeconomic groups consuming more healthy products (e.g. fruit and vegetables) and less unhealthy products (e.g. sugars and fats) [1–4]. Common explanations for these inequalities are differences in monetary resources [5], unequal access to healthy foods [6], or knowledge [7]. Indeed, these factors contribute to the explanation of inequalities in dietary intakes, but cannot fully explain the gradient in healthy intakes [8]. An additional, but far less mentioned mechanism is that of ‘social distinction’, whereby those in a higher socioeconomic position adopt dietary patterns by which they can distinguish themselves from lower socioeconomic groups [9–12]. Social distinction – most famously described by the French sociologist Pierre Bourdieu [9] as part of his notion of ‘cultural capital’ – may have become increasingly important as a determinant for health-related lifestyles, now that opportunities for distinction on the basis of material possessions have declined [13]. Due to increasing economic prosperity and wealth, more people are able to afford luxury consumer goods (e.g. smartphone ownership, branded clothing) and material conditions have become less important as a determinant of lifestyles [14]. Except for some theoretical contributions on this notion [12, 15], empirical studies focussing on the importance of social distinction for socioeconomic inequalities in dietary intake are scarce [10]. The rapid popularity of so-called ‘superfoods’ offers an interesting opportunity to do so.

Although no official guidelines exist to what constitutes a ‘superfood’, it usually applies to food products that contain high amounts of particular nutrients (e.g. anti-oxidants, vitamins, minerals), which were only recently marketed to a wider public in Western countries (e.g. spelt products, quinoa, goji berries). Given that the scientific evidence is inconclusive – there is no evidence that superfoods are healthier than equivalent alternatives – is must be merely the perception of health benefits that may have triggered the popularity. At the same time, high prices may have strengthened the exclusivity of the perceived super-healthy products. As such, superfoods consumption may be particularly prone to social distinction.

Building upon the theory of cultural capital, cultural participation (e.g. visiting museums, theatre) is arguably the best available indicator of distinction [9, 16, 17]. The knowledge and preferences that are required for the appreciation of cultural practices, such as paintings or ballet, are highly socially patterned and transmitted across generations [18, 19], making it a suitable method for distinction and exclusion [9]. Importantly, cultural participation is unlikely to be associated with the consumption of superfoods in a causal way (i.e. the mere act of a cultural visit is not likely to *cause* an increase in superfoods consumption). We therefore examined whether superfoods consumption is patterned by socioeconomic position and positively associated with this well-established indicator of social distinction. Specifically, we investigated whether the consumption of five popular and commonly available superfoods (i.e. spelt products, quinoa, goji berries, chia seeds and wheatgrass) is 1) more prevalent in high than low socioeconomic groups, and 2) associated with cultural participation, once adjusted for education, income, and potential confounders.

## Methods

Data were collected from participants of the Dutch population-based GLOBE study - a cohort study on socioeconomic inequalities in health in the Netherlands. The GLOBE study was initiated in 1991 when data was collected among a random sample of non-institutionalized persons (ages 15–74) from the city of Eindhoven and surrounding municipalities. More detailed information on the objectives, study design, and data collection of the Dutch GLOBE study can be found elsewhere [20, 21]. The use of personal data in the GLOBE study is in compliance with the Dutch Personal Data Protection Act and the Municipal Database Act, and has been registered with the Dutch Data Protection Authority (number 1248943).

For the purposes of this study, cross-sectional data from the fifth wave (2014) of the GLOBE study were used. Data were collected by means of a large-scale postal survey which was sent out to 10,668 persons, comprising 4,886 participants of the existing GLOBE cohort, supplemented with a random sample of 5782 newly selected persons from the municipality register of the city of Eindhoven. Of the respondents of the 2014 data collection (*N* = 4,851, response = 45.5%), those between 25 and 75 years old and living in the city of Eindhoven were selected to represent the target population. This resulted in a total sample of 2812 participants eligible for the study, among which 1114 participants from the original GLOBE cohort.

### Explanatory variables

Cultural participation was measured by asking respondents how often they visited seven cultural practices: art museums, historical museums, opera or ballet, classical concerts, theatre, architecture, and popular concerts or festivals (0 – never, 1 – once per year and 2 – more than once per year). These were summed and subsequently divided into quintiles, which resulted into categories that corresponded with the interpretation of one higher quintile equals 2 additional visits.

Highest attained educational level was classified according to the International Standard Classification of Education (ISCED): 1 – primary education (ISCED 0–1), 2 – lower secondary education (ISCED 2), 3 – upper secondary education (ISCED 3–4), 4 – tertiary education (ISCED 5–7).

Household equivalent income was measured as the level of household income per month divided by the square root of the number of people living from this income and divided into 5 categories: 1–< €1000/month, 2–€1000–€1500/month, 3–€1500–€2000/month, 4–€2000–2500/month and 5–> €2500/month.

Potential confounders included were sex (male, female), age (in 10-year age groups), living together with a partner (yes, no), country of birth (Netherlands, other), having children living at home (yes, no), employment status (full-time employed, part-time employed, unemployed, retired, homemaker, other), and weekly fruit and vegetable consumption (to adjust for ‘regular’ healthy food choices). Fruit and vegetable consumption was measured with a food frequency questionnaire [22]. Participants were asked how many times, on a weekly basis, they had consumed fruit and vegetables in the previous month. Subsequently they were asked to indicate how many portions they ate on a typical occasion. Consumption of fruit and vegetables was calculated by adding up how much fruit and vegetables (per 100 grams) participants consumed in total in a typical week, where 100 grams was used as the equivalent of one piece of fruit.

### Outcome variables

Participants were asked how many times per week on average they consumed five superfoods over the last month: spelt products, quinoa, goji berries, chia seeds and wheatgrass (selected because these are commonly available superfoods in The Netherlands). Because of the low prevalence of superfoods consumption, the variables were dichotomised (≥1/week, versus <1/week). The prevalence of goji berries, chia seeds and wheatgrass consumption were especially low. Since these products are usually consumed as added substances (e.g. to a salad or a smoothie), and may be consumed interchangeably (diluting the effect of the separate products), the three variables were summed in one variable that indicated consuming goji berries, chia seeds or wheatgrass at least once per week.

### Statistical analysis

First, descriptive statistics for the study sample were calculated. Second, age- and sex-standardized prevalence of superfoods consumption were calculated for the full sample and for all education, income and cultural participation groups. Third, multivariable logistic regression models were used to estimate the associations between each of the three explanatory variables separately (cultural participation, education and income) and the consumption of superfoods, adjusted for potential confounders. Fourth, a multivariable logistic regression model was used to estimate the associations between the explanatory variables and the consumption of superfoods adjusted for each other and potential confounders. Missing data were present for questions about living together with a partner (1.1%), country of birth (.5%), employment status (1.9%), fruit and vegetable consumption (3.3%), cultural participation (5.3%), educational level (.9%) and household equivalent income (12.7%) and were handled via multiple imputation (m = 5). Respondents with missing data on an outcome variable were excluded concerning that outcome variable, resulting in different samples for the three outcome variables: 1.9% missing on spelt products, 2.7% missing on quinoa and 2.3% missing on goji berries, chia seeds or wheatgrass. The analyses were weighted by respondent-level sample weights to account for the sampling strategy within the GLOBE study. All analyses were performed using Stata, version 14.

## Results

The mean age of the sample was 48.9 (SD 15.6) and 55.2% was female (Table 1). Prevalence rates of superfoods consumption ranged from 40.5% for spelt, 21.7% for quinoa, to 18.6% for goji berries, chia seeds or wheatgrass. Those with higher levels of education, income or cultural participation had higher prevalence rates of all superfoods (Table 2).

**Table 1:** Descriptive statistics of GLOBE 2014 participants^a^

Variables	Percentage or mean (SD)
Gender	
Men	44.8
Women	55.2
Age groups	
25–34 years	25.6
35–44 years	16.9
45–54 years	17.6
55–64 years	19.3
65–75 years	20.7
Living together with a partner	
Yes	74.0
No	26.0
Country of birth	
Netherlands	88.5
Else	11.5
Children living at home	
No	64.3
Yes	35.7
Employment status	
Full-time employed	38.8
Part-time employed	24.9
Unemployed	8.0
Retired	20.4
Homemaker	4.5
Other	3.4
Weekly intake of fruit & vegetables (per 100 grams)^b^	19.7 (10.0)
Cultural participation	
1 lowest (0–1 visits per year)	24.8
2 (at least 2 visits per year)	20.9
3 (at least 4 visits per year)	20.3
4 (at least 6 visits per year)	17.7
5 highest (at least 8 visits per year)	16.3
Educational level	
Primary	4.9
Lower secondary	20.9
Upper secondary	25.1
Tertiary	49.1
Household equivalent income	
< €1000/month	13.8
€1000–€1500/month	20.1
€1500–€2000/month	24.0
€2000–€2500/month	29.5
> €2500/month	12.7
Superfoods consumption (at least once per week)	
Spelt products	41.0
Quinoa	21.5
Goji berries, chia seeds or wheatgrass	18.6

^a^ Descriptive statistics calculated on non-imputed and weighted data

^b^ Weekly intake of fruit and vegetables is expressed as mean (standard deviation)

**Table 2:** Age- and sex-standardized prevalence of superfoods consumption by education, income and cultural participation

	Spelt products	Quinoa	Goji berries, chia seeds or wheatgrass
	At least once per week (%)	At least once per week (%)	At least once per week (%)
Total	40.5	21.7	18.6
Cultural participation			
1 lowest (0–1 visits per year)	23.5	7.8	11.3
2 (at least 2 visits per year)	34.4	14.8	16.3
3 (at least 4 visits per year)	41.3	22.0	17.2
4 (at least 6 visits per year)	53.2	31.4	23.0
5 highest (at least 8 visits per year)	59.8	38.1	30.8
Educational level			
Primary	19.1	7.7	12.9
Lower secondary	30.5	8.6	10.1
Upper secondary	35.0	15.4	16.9
Tertiary	50.3	30.9	21.9
Household equivalent income			
< €1000/month	29.9	15.3	18.5
€1000–€1500/month	36.9	15.8	16.4
€1500–€2000/month	40.7	20.8	18.1
€2000–€2500/month	48.0	27.7	20.1
> €2500/month	53.9	35.6	23.6

Cultural participation was strongly associated with the consumption of all superfoods, also when taking education and income into account (Table 3). Compared to participants in the lowest quintile of cultural participation, those in the highest quintile reported more consumption of spelt products (OR = 2.97, 95% CI = 2.10;4.18), quinoa (OR = 3.50, 95% CI = 2.12;5.79) and goji berries, chia seeds or wheatgrass (OR = 2.69, 95% CI = 1.73;4.17). The associations between education and income and superfoods consumption were strongly attenuated when adjusting for all variables (Table 3). Educational level remained associated with superfood consumption, although less strongly than cultural participation. Income was not independently associated with any of the outcomes. Associations of the potential confounders with superfood consumption are presented in Appendix 1.

**Table 3:** Multivariable logistic regression models of the association between cultural participation, educational level and income on superfoods consumption

		Spelt products (*N* = 2759)				Quinoa (*N* = 2737)				Goji berries, chia seeds or wheatgrass (*N* = 2746)			
		Crude model^a^		Adjusted model^b^		Crude model^a^		Adjusted model^b^		Crude model^a^		Adjusted model^b^	
Variables	Categories	OR	95% CI	OR	95% CI	OR	95% CI	OR	95% CI	OR	95% CI	OR	95% CI
Cultural participation (quintiles)	1 lowest	1		1		1		1		1		1	
	2	1.54	(1.16, 2.05)	1.39	(1.04, 1.87)	1.64	(1.06, 2.55)	1.40	(.89, 2.22)	1.53	(1.03, 2.29)	1.49	(.98, 2.25)
	3	1.99	(1.48, 2.67)	1.72	(1.25, 2.35)	2.68	(1.72, 4.16)	2.08	(1.29, 3.35)	1.59	(1.07, 2.36)	1.55	(1.01, 2.36)
	4	3.06	(2.26, 4.15)	2.54	(1.83, 3.52)	4.09	(2.64, 6.34)	2.94	(1.83, 4.72)	2.04	(1.37, 3.04)	1.98	(1.28, 3.05)
	5 highest	3.72	(2.72, 5.08)	2.97	(2.10, 4.18)	5.37	(3.41, 8.47)	3.50	(2.12, 5.79)	2.83	(1.89, 4.22)	2.69	(1.73, 4.17)
Educational level	Primary	1		1		1		1		1		1	
	Lower secondary	2.15	(1.23, 3.76)	1.78	(1.00, 3.16)	1.13	(.46, 2.81)	.91	(.36, 2.33)	1.19	(.56, 2.53)	1.10	(.51, 2.40)
	Upper secondary	2.58	(1.46, 4.54)	1.87	(1.04, 3.37)	2.12	(.88, 5.11)	1.45	(.58, 3.63)	1.79	(.85, 3.76)	1.50	(.69, 3.27)
	Tertiary	4.36	(2.49, 7.63)	2.55	(1.41, 4.62)	4.70	(1.97, 11.25)	2.37	(.93, 6.02)	2.08	(1.00, 4.32)	1.50	(.68, 3.32)
Household equivalent income	<€1000	1		1		1		1		1		1	
	€1000–€1500	1.20	(.85, 1.69)	1.02	(.71, 1.45)	.84	(.53, 1.33)	.71	(.44, 1.15)	.82	(.51, 1.31)	.73	(.44, 1.20)
	€1500–€2000	1.34	(.93, 1.93)	.97	(.67, 1.41)	1.15	(.72, 1.84)	.81	(.50, 1.31)	.87	(.56, 1.34)	.69	(.44, 1.09)
	€2000–€2500	1.64	(1.16, 2.31)	.97	(.66, 1.42)	1.58	(1.01, 2.46)	.87	(.55, 1.40)	.94	(.61, 1.45)	.68	(.42, 1.07)
	>€2500	1.93	(1.28, 2.91)	1.01	(.65, 1.58)	2.48	(1.53, 4.01)	1.18	(.71, 1.95)	1.07	(.65, 1.77)	.71	(.41, 1.22)

^a^All models were adjusted for confounders: sex, age, living together with a partner, country of birth, children living at home and employment status and fruit and vegetable consumption

^b^All models included cultural participation, educational level, household equivalent income, sex, age, living together with a partner, country of birth, children living at home, employment status and fruit and vegetable consumption

## Discussion

This is the first study to show that the consumption of five popular ‘superfoods’ was highly patterned by socioeconomic position. Superfoods consumption was also strongly correlated with cultural participation – a classical indicator of social distinction – independent of education, income, and a range of potential confounders. Moreover, the associations between education and income and superfoods consumption were strongly attenuated after inclusion of indicators of cultural participation.

To the best of our knowledge, no previous studies have tried to link a well–established indicator of social distinction to the consumption of superfoods in a large population-based cohort. Yet, our study suffers from some limitations. First, cultural participation and superfoods consumption were self-reported. Although these questions were only a small part of a broader questionnaire, it may be that higher socioeconomic groups are more likely to report higher levels of cultural participation and superfoods consumption due to social desirability (as a result of the hypothesised distinction mechanism). Second, we only studied five types of ‘superfoods’. Even though these superfoods are among the most readily available in The Netherlands, they constitute a limited number of potential superfood products. Third, we also included a limited number of cultural activities, but there are likely more social distinction instruments. For instance, a previous study examined whether smoking can be seen as a form of distinction by testing the relationship between musical tastes and smoking [23]. In our study we chose cultural participation as a more suitable indicator of distinction, since a recent review found that this indicator was most often used to measure ‘embodied cultural capital’ [16]. In line with the work of Pierre Bourdieu, it is embodied cultural capital (internalized dispositions, such as skills, tastes and attitudes that can be used for distinction and exclusion) [9, 24, 25] that is most relevant for distinctive lifestyle patterns [15]. Fourth, we tried to adjust for several important confounders. For instance, availability of time to participate in cultural activities was controlled for by including employment status and having children living at home [26]. Also, sensitivity analyses showed that controlling for social participation (frequency of participating in several associations and organizations, e.g. neighbourhood association, political organization, sport club, volunteer organization) did not change the findings. However, we cannot rule out that there are other determinants that are related to both cultural participation and the consumption of superfood for which we did not control. For instance, certain personality traits (e.g. openness to experience) could make it more likely that someone visits cultural activities and consumes superfoods [16].

Our findings are in line with previous findings that higher socioeconomic groups make more health-conscious food choices than lower socioeconomic groups [27]. The early adoption of innovative products in higher socioeconomic groups is also seen for other products [28]. The clear association with cultural participation however, adds to this that social distinction may be an important underlying mechanism. Because superfoods are more expensive and exclusive, they may be attractive as a means to distinguish oneself from other social groups [10, 29]. Yet, the strong attenuation of the observed association between income and superfoods consumption also suggests that income in itself is not an important determinant of superfoods intake. Future research should examine whether distinction is indeed also relevant for more common dietary products with established health benefits and relative high prices, and whether the socioeconomic gradient in healthy dietary intake is to some extent reflective of this process. For these purposes, it could be worthwhile to use methods that are able to measure the mechanism of distinction (both implicit and explicit) more directly (e.g. Implicit Association Tests [30] or prototype perceptions [31]).

An important implication from our study is that interventions aimed at improving dietary choices in lower socioeconomic groups particularly, should be aware of the mechanism of social distinction. Distinction reflects a lifelong socialization process that is influenced by the norms, habits and preferences within socio-cultural environments [9, 12, 15, 32]. Dietary patterns are part of a distinct lifestyle pattern and some low socioeconomic subgroups may be more inclined to consume foods that are cheap and high in fat, and may be less concerned with healthiness than their advantaged counterparts [9, 27, 29, 33]. Therefore, it can be particularly challenging to improve the dietary behaviors of socioeconomically disadvantaged people [34]. Interventions focussing on young children and their parents may therefore be most successful, as incorporating healthy food habits may then become part of the socialisation process. On the other hand, interventions may also aim to reduce the exclusiveness of healthy products, and thus change the socio-cultural meaning and ‘distinctive value’ of healthy food products.

## Conclusions

The consumption of ‘superfoods’ seems to be a contemporary expression of social distinction in higher socioeconomic groups. This distinction mechanism may be an important determinant of inequalities in dietary intake. It deserves a more prominent role in interventions to reduce these inequalities.

## Appendix

**Table 4:** Multivariable logistic regression models of the association between the explanatory variables and the consumption of superfoods

		Spelt products			Quinoa			Goji berries, chia seeds or wheatgrass		
Variables	Categories	OR	95% CI		OR	95% CI		OR	95% CI	
Sex	Men	1			1			1		
	Women	1.89	1.54	2.31	2.06	1.62	2.62	2.14	1.67	2.74
Age groups	25–34	1			1			1		
	35–44	1.06	.80	1.41	.71	.51	.99	.74	.53	1.04
	45–54	1.25	.93	1.69	.90	.63	1.28	.57	.39	.82
	55–64	.97	.72	1.31	.65	.45	.93	.57	.40	.82
	65–75	.77	.44	1.32	.46	.21	1.00	.30	.13	.68
Living together with a partner	Yes	1			1			1		
	No	.98	.77	1.25	.98	.73	1.31	1.01	.76	1.36
Birth country	The Netherlands	1			1			1		
	Else	.73	.54	1.00	.83	.57	1.20	1.65	1.17	2.34
Children living at home	No	1			1			1		
	Yes	.83	.65	1.06	.89	.67	1.19	.97	.72	1.32
Employment status	Full-time employed	1			1			1		
	Part-time employed	1.07	.83	1.38	.96	.72	1.28	.98	.73	1.32
	Unemployed	.52	.34	.79	.60	.36	.99	.79	.48	1.31
	Retired	1.28	.74	2.21	.61	.28	1.31	.84	.37	1.90
	Non-employed	.94	.56	1.57	.57	.27	1.16	.65	.33	1.25
	Other	1.00	.62	1.59	1.08	.61	1.90	1.21	.68	2.16
Weekly fruit & vegetable consumption (per 100 grams)		1.03	1.02	1.04	1.03	1.02	1.05	1.05	1.04	1.06
Cultural participation (quintiles)	1 lowest	1			1			1		
	2	1.39	1.04	1.87	1.40	.89	2.22	1.49	.98	2.25
	3	1.72	1.25	2.35	2.08	1.29	3.35	1.55	1.01	2.36
	4	2.54	1.83	3.52	2.94	1.83	4.72	1.98	1.28	3.05
	5 highest	2.97	2.10	4.18	3.50	2.12	5.79	2.69	1.73	4.17
Educational level	Primary	1			1			1		
	Lower secondary	1.78	1.00	3.16	.91	.36	2.33	1.10	.51	2.40
	Upper secondary	1.87	1.04	3.37	1.45	.58	3.63	1.50	.69	3.27
	Tertiary	2.55	1.41	4.62	2.37	.93	6.02	1.50	.68	3.32
Household equivalent income	<€1000	1			1			1		
	€1000–€1500	1.02	.71	1.45	.71	.44	1.15	.73	.44	1.20
	€1500–€2000	.97	.67	1.41	.81	.50	1.31	.69	.44	1.09
	€2000–€2500	.97	.66	1.42	.87	.55	1.40	.68	.42	1.07
	>€2500	1.01	.65	1.58	1.18	.71	1.95	.71	.41	1.22

All models included cultural participation, educational level, household equivalent income, sex, age, living together with a partner, country of birth, children living at home, employment status and fruit and vegetable consumption
